# EpiFloripa Aging cohort study: methods, operational aspects, and follow-up strategies

**DOI:** 10.11606/S1518-8787.2017051006776

**Published:** 2017-11-13

**Authors:** Ione Jayce Ceola Schneider, Susana Cararo Confortin, Carla de Oliveira Bernardo, Carolina Carvalho Bolsoni, Danielle Ledur Antes, Karine Gonçalves Pereira, Lariane Mortean Ono, Larissa Pruner Marques, Lucélia Justino Borges, Maruí Weber Corseuil Giehl, Rodrigo de Rosso Krug, Vanessa Fernanda Goes, Alexandra Crispim Boing, Antônio Fernando Boing, Eleonora d’Orsi

**Affiliations:** I Departamento de Ciências da Saúde. Universidade Federal de Santa Catarina. Araranguá, SC, Brasil; II Programa de Pós-Graduação em Saúde Coletiva. Universidade Federal de Santa Catarina. Florianópolis, SC, Brasil; III Universidade Federal de Santa Catarina. Florianópolis, SC, Brasil; IV Complexo de Ensino Superior Meridional. Passo Fundo, RS, Brasil; V Programa de Pós-Graduação em Ciências Médicas. Universidade Federal de Santa Catarina. Florianópolis, SC, Brasil; VI Departamento de Educação Física. Universidade Federal do Paraná. Curitiba, PR, Brasil; VII Programa de Pós-Graduação em Nutrição. Universidade Federal de Santa Catarina. Florianópolis, SC, Brasil; VIII Departamento de Saúde Pública. Universidade Federal de Santa Catarina. Florianópolis, SC, Brasil

**Keywords:** Health of the Older Adults, Health Surveys, methods, Interviews as Topic, utilization, Sampling Studies, Data Collection, methods, Saúde do Idoso, Inquéritos Epidemiológicos, métodos, Entrevistas como Assunto, utilização, Amostragem, Coleta de Dados, métodos

## Abstract

**OBJECTIVE:**

To describe the sample plan, operational aspects, and strategies used in the 2009/2010 and 2013/2014 EpiFloripa Aging Study.

**METHODS:**

The EpiFloripa Aging is a population-based longitudinal study with 1,705 older adults (60 years or more) living in the municipality of Florianópolis, State of Santa Catarina, Brazil, in 2009/2010 (baseline). The research was conducted with a face-to-face interviews, organized into blocks of identification, socioeconomic, mental health, health and life habits, global functionality, falls, physical activity, morbidities, use of health services, use of medications, food, oral health, and violence, evaluated in the first (2009/2010) and in the second wave (2013/2014). Additionally, in the second wave, we investigated the issue of discrimination and quality of life.

**RESULTS:**

The response rate of the first wave was 89.2% (n = 1,705). The baseline sample, with predominance of women (63.9%), was similar to the 2010 Census regarding age for women and slightly different for younger men. In the second wave, 1,197 participants were interviewed (response rate of 70.3%). Follow-up losses were only observed for the variable age group (p = 0.003), and predominantly for those aged 80 years or more. Mortality data linkage and active search for participants were used as a follow-up strategies.

**CONCLUSIONS:**

This study used strategies that were able to help locate the participants and maintain adherence, which ensured a good response rate during investigations.

## RESUMO

**OBJETIVO:**

Descrever o plano amostral, aspectos operacionais e estratégias utilizadas no Estudo EpiFloripa Idoso 2009/2010 e 2013/2014.

**MÉTODOS:**

O EpiFloripa Idoso é um estudo de base populacional com 1.705 idosos (60 anos ou mais) residentes no município de Florianópolis, SC, em 2009/2010 (linha de base). A investigação foi realizada por meio de entrevista face a face, organizada nos blocos de identificação, geral (características socioeconômicas), saúde mental, saúde e hábitos de vida, funcionalidade global, quedas, atividade física, morbidades, uso de serviços de saúde, uso de medicamentos, alimentação, saúde bucal e violência, avaliados na primeira (2009/2010) e na segunda onda (2013/2014). Adicionalmente, na segunda onda, investigou-se a temática de discriminação e qualidade de vida.

**RESULTADOS:**

A taxa de resposta na primeira onda foi de 89,2% (n = 1.705). A amostra da linha de base, com predomínio de mulheres (63,9%), foi semelhante ao Censo 2010 em relação à idade nas mulheres e ligeiramente diferente nos homens mais jovens. Na segunda onda, 1.197 participantes foram entrevistados (taxa de resposta de 70,3%). Houve perda de seguimento somente para a variável faixa etária (p=0,003), principalmente naqueles com 80 anos ou mais. Utilizou-se como estratégias de seguimento o relacionamento de dados e a busca ativa.

**CONCLUSÕES:**

O presente estudo utilizou estratégias que conseguiram auxiliar na localização dos participantes e manutenção da aderência, o que garantiu boa taxa de resposta durante as investigações.

## INTRODUCTION

Cohort studies have been present in the area of epidemiology for decades. These studies allow researchers to know, besides the incidence of health problems in the population, the causal relationship between outcomes and several factors, such as the demographic, social, economic, and behavioral ones. The longitudinal design allows the exclusion of reverse causality^9^ and allows the reduction of recall bias. On the other hand, there may be loss of follow-up, to be avoided with the use of strategies to locate and motivate the participants^8^.

Cohort studies with older adults are mainly aimed at investigating predictors of adverse health events in this age group, such as chronic and multiple diseases, which persist for several years, or acute disabling events^13^ ^,^ ^14^ ^,^ ^19^. Among the cohorts with older adults carried out in Brazil, we can mention the SABE Study (Health, Welfare, and Aging – São Paulo)^14^, the Bambuí Project (Minas Gerais)^13^, and the Epidoso (Epidemiology of the Older Adult – São Paulo)^19^.

These cohort studies result in the publication of, almost exclusively, their results. Thus, we considered as relevant, in order to improve future studies, the description of the operational aspects, the provision of data on the main obstacles faced, the efficient solutions, and the best practices for population-based household surveys. In this case, with older adults, we have complications of another order, among them, dropouts because of deaths and incapacitating diseases.

In Florianópolis, State of Santa Catarina, Brazil, the EpiFloripa Aging cohort study sought to investigate the health conditions of older adults living in the urban area of the municipality. The study began in 2009 and results of cross-sectional analyses in the first wave of the study have already been published with different outcomes (www.epifloripa.ufsc.br). This article aims to describe the methods and strategies used in the EpiFloripa Aging *S*tudy in 2009/2010 and 2013/2014.

## METHODS

This is a prospective, population-based, home-based cohort study entitled “EpiFloripa: health conditions of older adults in Florianópolis”. The first wave (baseline) happened in 2009/2010 and the second wave in 2013/2014.

The EpiFloripa Aging Study was carried out in the urban area of Florianópolis, State of Santa Catarina, located in the southern region of Brazil. The population estimated by the Brazilian Institute of Geography and Statistics (IBGE)^a^, for 2009, was 408,163 inhabitants, of whom 10.9% belonged to the age group of 60 years or more. In the 2010 Census^b^, population was 433,158 inhabitants, being 11.5% aged 60 years or more. Life expectancy at birth in 2010 was 77.4 years, and according to the United Nations Development Programme in Brazil^c^, the Municipal Human Development Index (MHDI) was 0.847, which is the third position among Brazilian municipalities and the first one among Brazilian capitals.

### Definition of Study Population and Sampling

The study population consisted of older adults (60 years old or more) living in the urban area of Florianópolis in 2009/2010. As the objective of the study was to investigate different health outcomes, we considered for the calculation of the sample size the expected prevalence of 50%, a four-point error, and 95% confidence interval (95%CI), and design effect (*deff*) for samples by conglomerates estimated as equal to two. We added 20% for expected losses and 15% to test associations. We took into account the size of the population aged 60 or more, equal to 44,460 persons, referring to the population estimate for 2009. According to the sample calculation, the minimum number of interviews should be 1,599.

The sample selection process was carried out by conglomerates in two steps. The units of the first step were the census tracts (IBGE census units) and the units of the second step were the households.

According to the 2000 IBGE Census, the municipality consisted of 460 census tracts (429 urban, 28 rural, 2 isolated urban, and 1 urban-*favela* extension). Only urban census tracts were included. From the 429 urban census tracts, nine were excluded because they were non-domiciles. These 420 census tracts were ordered according to the average monthly income of the head of the family (R$314.76 to R$5,057.77) and 80 of them were systematically drawn.

After obtaining the maps of the census tracts (http://mapas.ibge.gov.br/bases-e-referenciais/bases-cartograficas/malhas-digitais.html), the number of households was updated (listing). In addition to updating the number of households, we verified the characteristics of the sector and the search for partnerships in regions considered as a security risk. The main partnership took place with the Local Health Units and Family Health Strategy Units of the Municipal Health Department (SMS) of Florianópolis.

The number of households per tract ranged from 61 to 725. Those with less than 150 households were grouped, when geographically close, and the tracts with more than 500 households were divided in order to reduce the coefficient of variation of the number of households, from 52.7% (n = 80) to 35.2% (n = 83). This resulted in 83 tracts for data collection, consisting of 22,846 permanently occupied households.

The strategy of working with conglomerates by census tract was advantageous, since they cover the entire population and territory under study, have clear and well defined territorial boundaries, have size with objectives of sample composition, and have enough amount of representative numbers of internal population.

According to data from the 2010 IBGE Census, the average number of residents per household in Florianópolis was 3.1 persons. As the population targeted by the survey corresponded to, approximately, 11% of the population, an average of 102 persons in the age group of interest were found per census tract, or one in every three households. Therefore, approximately 60 households should be visited per census tract to find 20 older adults. To this end, households were systematically drawn and all older adults living in the households chosen were considered as eligible for the survey.

Because of the availability of financial resources, the number of older adults interviewed per tract increased to 23, in order to increase the variability of the sample. Thus, 1,911 eligible older adults were found in the randomly selected households.

### Inclusion and Exclusion Criteria

In the first wave, individuals should be aged 60 years or more (completed in the year of the survey). As exclusion criteria, we considered the institutionalized older adults (long-term institutions, hospitals, and penitentiaries).

In the second wave, the inclusion criterion was to have been interviewed in 2009/2010. Exclusion criteria were duplicity and incompatible age. To maintain follow-up, the older adults who moved to long-term institutions were interviewed with prior authorization from the family member responsible for them and the consent of the institution and the older adult.

### Identification of Deaths

Before contacting the older adults participating in the first wave of the study (baseline – 2009/2010), we sought to identify deaths, date and basic cause, based on the Declaration of Death. This search was performed in the database of the Mortality Information System (SIM) of the Ministry of Health, referring to persons aged 60 years or more from Santa Catarina, at two moments: at the beginning and at the end of the interview cycle. The SIM database was obtained by request from the State Health Department of Santa Catarina.

After the first search in the SIM database, the older adults not identified as dead were contacted (telephone, e-mail, letter etc.) for confirmation of vital status and data update.

The elderly who did not have a contact telephone number were searched directly at the addresses of the data collection of 2009/2010. Unique cases in which there was no telephone and address record were investigated in the systems used by the Local Health Units of Florianópolis (InfoSaúde System), social networks, telephone directory, and neighbors. The address and phone numbers of 1,141 older adults were updated and confirmed.

Deaths identified during data collection were recorded according to the data provided by family members or guardians, and their information was confirmed, when possible, from a second consultation to the SIM database, performed at the end of the collection.

To facilitate and systematize the search for the deaths in the SIM database, we used the technique of statistical probabilistic linkage of records^6^, in the program OpenRecLink^®^, version 2.8 (http://reclink.sourceforge.net/).

From the intersection between the EpiFloripa database and the SIM database, 200 deaths were found. By telephone contact, another 17 deaths were identified, which amounted to 217 deaths.

### Research Instrument

The research instrument, applied as an interview, was elaborated during weekly meetings by a group of professors, undergraduate and graduate students of the Universidade Federal de Santa Catarina (UFSC), involved in the operationalization of the EpiFloripa Aging.

The weekly meetings of the first wave (March to September 2009) and second wave (April to November 2013) allowed for discussions and reflections on the maintenance, exclusion, or inclusion of scales. As an advantage of this collective work, we can highlight the involvement and participation of the group in the decisions, generating motivation and commitment. However, it can be difficult to take into account all interests because of the number of questions in the instrument that impact on the longer duration of the interview.

As soon as the first version of the questionnaire was defined, a professional with systems technology training, in the first wave, and an undergraduate student in Computer Science, in the second wave, were hired to carry out the questionnaire programming.

In the first wave (2009/2010), the instrument had 306 questions organized into 13 blocks (http://www.epifloripa.ufsc.br/wp-content/uploads/2011/06/QUESTIONARIO_IDOSO-20091.pdf). The general block consisted of demographic and socioeconomic information, collected based on the IBGE. The mental health part involved the assessment of cognitive status, investigated by the Mini-Mental State Examination^11^ and categorized according to Almeida^1^, and screening for depressive symptoms^24^.

Regarding health and life habits, the questionnaire covered the perception of health status^23^, smoking (according to questions from the National Household Sample Survey – PNAD)^d^, alcohol consumption^2^, and global functionality^10^ ^,^ ^21^. The anthropometric data of height and weight were measured, respectively, by portable stadiometer and digital scale with resolution of 100 grams. To measure waist circumference, we used a flexible and inextensible metric tape, and the measurement was performed on the narrowest part of the trunk below the last rib^12^. Blood pressure was measured in the wrist (distance of two centimeters from the radioulnar joint) by a digital Techline^®^ sphygmomanometer, dully calibrated.

The self-report of morbidities was recorded based on the National Household Sample Survey (*Pesquisa Nacional por Amostra de Domicílios* – PNAD) and chronic pain questions. In addition, we evaluated the use of health services, women’s health^16^, oral health (with questions based on the PNAD^e^ ^,^ ^f^ and the Brazilian Oral Health Survey^g^), diet^17^, physical activity in the leisure and transportation domains^4^ ^,^ ^7^, and perception of the environment^22^, in addition to information about drug use and risk of violence against the older adult^h^.

In the second wave (2013/2014), the instrument maintained the organization of the first one, in addition to including questions about discrimination^3^, quality of life (CASP-19)^15^, fear of falls^5^, and domestic violence^18^ ^,^ ^20^, and questions such as health of women and use of health services were excluded, among others. Therefore, the second wave questionnaire was structured into 655 questions (15 blocks) (http://www.epifloripa.ufsc.br/wp-content/uploads/2013/07/EpiFloripaIdoso_2013_QUESTIONARIO.pdf).

### Fieldwork Logistics

The coordinators and supervisors of the study carried out the selection and training of the interview team. A manual was developed to deepen the knowledge about the research and the collection instrument, to facilitate the interviewer-interviewed interaction, and to clarify doubts about the application of the questionnaire.

The interviewers were previously trained in two steps, one showing the project and the instrument, and another on the field. This process was performed in the two waves for instrument testing and refinement and calibration of the tests.

In the first wave, personal digital assistants (PDA), provided by IBGE, were used, which allowed the direct export of the information for the construction of the database, which allowed us to skip the typing step. In the second wave, we chose to use a netbook because of the ease of handling for the application of the questionnaire.

### Pilot Study

The pilot study of the first wave was carried out in August 2009, in random census tracts to compose the sample. We interviewed 99 older adults, 56.8% female, with a mean age of 70.68 years. In 2013/2014, the pilot study was carried out between October and November 2013. We interviewed 70 older adults (46 women), with a mean age of 71.1 years.

### Data Collection

The fieldwork of the first wave was carried out between September 2009 and June 2010, amounting to 10 months of collection. The relatively long time of collection was due to the difficulty in finding the older adults, the availability to carry out the interviews, and, mainly, the small number of interviewers in the field. Each interviewer received the map of the tract that they had to go through, as well as the list of households that had been randomly selected. The interviewer went to each household to identify eligible individuals and to schedule the interviews.

The second wave of the study occurred from November 2013 to October 2014. The supervisors recommended scheduling the interview in advance. However, for the older adults who could not be reached by phone, the interviewers were asked to visit the residence. At the end of each interview, security measures such as saving and automatic storage of files over the Internet were used to prevent loss and facilitate the process of saving the data.

### Losses and Refusals

We considered as losses older adults who were not located after four home visits, being at least one at the weekend and one at night in the two waves, and those not located in 2013/2014. In the second wave, the older adults who were hospitalized for the entire period of collection were considered as lost. Those who expressed no interest in participating were considered as refusals.

### Technical Support

Weekly meetings of the team were carried out to monitor the interviews and discuss situations that occurred during the interviews, strategies to approach refusals, and reports on the progress of the collection, aiming to solve problems and difficulties encountered in the fieldwork.

### Data Consistency Analysis

Data consistency analysis was performed in all interviews, both in 2009/2010 and in 2013/2014, comparing the simple frequency of the data with what was expected. Incongruent responses were rectified from the clarification with the participant.

### Quality Control

In 2009/2010, the quality control of the interviews was carried out by the application of a questionnaire with 13 questions in approximately 10% of the interviewees, via telephone. In 2013/2014, a questionnaire with eight questions was applied, following the 2009/2010 standard. The Kappa test was applied to calculate the reproducibility of the questions. Kappa values ranged from 0.30 to 0.90 in the first wave, and from 0.50 to 0.94 in the second wave. Most questions had good to excellent reproducibility.

### Ethical Aspects

The EpiFloripa Aging project complied with all ethical precepts, according to Resolution 196 of 1996, of the Brazilian Health Council (CNS), in effect at the time of the first wave, approved by the Research Ethics Committee with Humans (CEPSH) of UFSC, protocol 352/2008. In 2013/2014, it was approved by the Ethics Committee under CAAE 16731313.0.0000.0121, and the principles of Resolution CNS 466 of December 12, 2012, were respected. All participants who agreed to participate in the research signed the informed consent.

### Financing and Accountability

In 2009/2010, funding was obtained from the National Council for Scientific and Technological Development (CNPq), Notice 06/2008 *Faixa B* (Project 569834/2008-2), in the total amount of R$59,000.00, in addition to partnerships with UFSC, IBGE, and SMS of Florianópolis. Of the financing, 85.0% was used to pay for interviews and 15.0% for the purchase of permanent materials.

In 2013/2014, the research did not obtain funding and it was made possible by the partnerships established with UFSC and other research projects, in addition to the assistance of students and professors. The FIOCRUZ lent the netbooks used in the fieldwork.

## RESULTS

In the households chosen from the baseline, 1,911 eligible older adults were found, and 1,705 were interviewed (89.2% response rate). The distribution of the response rate per income decile is shown in the Figure. Only decile 10 presented a response rate below 80.0%.

**Figure:**
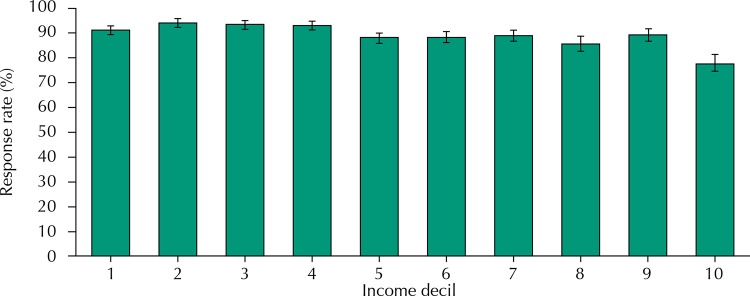
Response rate per decile of average income of the head of the family of the census tract. EpiFloripa Aging, Florianópolis, State of Santa Catarina, Brazil, 2000.

Table 1 shows the percentage distribution according to age group and sex of the participants of the EpiFloripa Study in comparison with the 2010 IBGE Census. There was little difference for the age groups of 60–64, 70–74, and 75–79 years for women, whereas there were differences in those aged 60–64 and 75–79 years for men.

**Table 1:** Comparison of the percentage distribution of older adults according to age groups, by gender, of the 2010 IBGE Census and the Study EpiFloripa Aging.

Age group (years)	Female		Male	
	
	IBGE	EpiFloripa	IBGE	EpiFloripa
			
	%	% (95%CI)	%	% (95%CI)
60 to 64	32.3	27.0 (23.9–30.4)*	37.2	29.8 (24.8–35.5)*
65 to 69	21.9	21.6 (18.7–24.9)	24.0	24.1 (20.4–28.1)
70 to 74	16.6	20.0 (17.7–22.6)*	17.4	18.7 (15.4–22.5)
75 to 79	12.5	16.5 (13.8–19.5)*	10.9	15.3 (11.6–20.0)*
80 to 84	8.9	8.3 (6.1–11.2)	6.6	7.2 (5.2–9.9)
85 to 89	4.9	4.0 (2.9–5.4)	2.8	3.4 (2.1–5.4)
90 to 94	2.0	2.1 (1.1–3.9)	0.9	0.7 (0.2–2.1)
95 to 99	0.6	0.3 (0.1–0.8)	0.2	0.6 (0.2–2.1)
100 or more	0.1	0.2 (0.0–0.6)	0	0.1 (0.0–0.9)

* Value significantly different from the 2010 IBGE Census data.

Table 2 shows the demographic characteristics and the baseline health conditions and follow-up. Three cases were excluded, two because they were duplicates and one because the age was incompatible with the study. Thus, 1,702 persons were considered for the first wave, with a median age of 70 years (range: 60–104 years). Regarding the first wave, the distribution of sociodemographic variables showed a predominance of women (63.9%), being almost half (49.8%) of the participants aged 60–69 years, with a high percentage of subjects with low education level (44.4% had less than four years of study). More than half (58.2%) was married. Regarding the selected health conditions, approximately a fourth of the older adults (26.3%) had probable cognitive impairment and almost one third (31.6%) reported dependence on four or more activities of daily living.

**Table 2:** Description of the sample interviewed in 2009/2010, and follow-up in 2013/2014. EpiFloripa Aging, Florianópolis, State of Santa Catarina, Brazil.

Variable	Response rate				p

	First wave		Second wave		
	
	n	%	n	%	
Sex					0.553
Female	1,088	63.9	778	71.5	
Male	614	36.1	419	68.2	
Total	1,702	100	1,197	70.3	
Age group (years)					0.003
60 to 69	848	49.8	642	75.7	
70 to 79	615	36.1	436	70.9	
80 or more	239	14.0	119	49.8	
Total	1,702	100	1,192	70.0	
Marital status					0.740
Married	990	58.2	718	72.5	
Single	99	5.8	72	72.7	
Divorced/Separated	132	7.8	88	66.7	
Widow	481	28.3	319	66.3	
Total	1,702	100	1,197	70.3	
*Per capita* income in quartiles					0.753
Lower quartile	426	25.0	298	70.0	
2nd Quartile	435	25.6	288	66.2	
3rd Quartile	424	24.9	301	71.0	
Higher Quartile	417	24.5	310	74.3	
Total	1,702	100	1,197	70.3	
Education level					0.445
No education	158	9.3	93	58.9	
1 to 4 years	595	35.1	430	72.3	
5 to 8 years	307	18.1	199	64.8	
9 to 11 years	241	14.2	180	74.7	
12 years or more	339	23.2	292	86.1	
Total	1,694	100	1,194	70.5	
Cognitive impairment^a^					0.923
Absence of probable cognitive impairment	1,244	73.7	875	70.3	
Presence of probable cognitive impairment	443	26.3	309	69.8	
Total	1,690	100	1,184	70.1	
Dependence on Activities of Daily Living (ADL)^b^					0.068
Zero	457	26.9	336	73.5	
1 to 3	708	41.6	531	75.0	
4 or more	537	31.6	330	61.5	
Total	1,702	100	1,197	70.3	

^a^ Investigated by the Mini-Mental State Examination^11^, categorized according to Almeida^1^.

^b^ Evaluated using the Brazilian Multidimensional Functional Assessment Questionnaire adapted from the questionnaire Old Americans Resources and Services (BOMFAQ/OARS), categorized according to Rosa et al.^21^

In the second wave, 1,197 (70.3%) persons were interviewed, as there were 217 deaths (12.8%), 159 losses (9.3%), and 129 refusals (7.6%). The median age of the respondents was 73 years (range: 63 to 107 years). Follow-up losses, of the selected variables, were only observed for the variable age group (p = 0.003), and predominantly for 80 years or more (Table 2).

## FINAL CONSIDERATIONS

The EpiFloripa is one of the few population-based cohort studies with older adults in Brazil with the objective of knowing and monitoring the health conditions of this population. It also aims to contribute with the development of health policies and actions for older adults.

In addition to the diffusion of knowledge, this work contributes with the development of research studies and qualification of human resources at the *stricto sensu* graduate level, from the participation in the research team, sharing of strategies used against the obstacles, and solutions for their transposition.

As the main challenges faced for the follow-up of this population, we can mention dropouts by deaths and changes of address; however, changing of addresses can be solved with continuous contact with the research participants. In addition, the increasing availability of epidemiological databases, coupled with the use of data linkage techniques, represents a viable alternative for follow-up.

In the second wave, there was the concern about not extending field collection for a long period, given the possibility of losing the older adult in the follow-up. However, the achievement of the goal was hindered by turnover, given the temporary nature of the work as interviewer and the difficulty in finding some persons because of the changes in address.

As for the methodological limitations, the study was faced with interviewer turnover and need for replacement. Some instruments used may have generated recall bias and the extension of the questionnaire. However, the study was well accepted by the participants. On the other hand, a positive strategy was the involvement of the team and the active participation in the decisions during all stages of EpiFloripa. The commitment and motivation of the EpiFloripa team is decisive for the successful conduction of the study and they may be related to the possible development of master’s and doctoral thesis with the study data.

Despite the challenges encountered in transforming the cross-sectional study into a cohort, EpiFloripa continues to follow 70.3% of its original sample. The results obtained subsidize improvements in public policies related to care for older adults and contribute with the structuring and sedimentation of a new cohort in Brazil. The consolidation of the EpiFloripa is of great value in the national scenario, since it portrays the older population living in a municipality with characteristics that are different from those where other cohorts in the country occur, considering its high MHDI.

## Footnotes

a Instituto Brasileiro de Geografia e Estatística. Estimativas populacionais para os municípios brasileiros. Rio de Janeiro: IBGE; 2009 [cited 2017 Aug 11]. Available from: http://www.ibge.gov.br/home/estatistica/populacao/estimativa2009/default.shtm

b Instituto Brasileiro de Geografia e Estatística. Censo demográfico 2010. Rio de Janeiro: IBGE; 2011 [cited 2017 Aug 11]. Available from: http://www.ibge.gov.br/home/estatistica/populacao/censo2010/default.shtm

c Programa das Nações Unidas para o Desenvolvimento no Brasil; Instituto de Pesquisa Econômica Aplicada; Fundação João Pinheiro. Atlas de desenvolvimento humano no Brasil: perfil: Florianópolis, SC. Brasília (DF): PNUD Brasil; 2013 [cited 2017 Aug 11]. Available from: http://www.atlasbrasil.org.br/2013/pt/perfil_m/florianopolis_sc

d Instituto Brasileiro de Geografia e Estatística. Pesquisa Nacional por Amostra de Domicílios - PNAD 2008. Questionário da pesquisa. Rio de Janeiro: IBGE; 2008 [cited 2017 Jun 25]. Available from: http://www.ibge.gov.br/home/estatistica/populacao/trabalhoerendimento/pnad2008/suplementos/tabagismo/qstpnad2008.pdf

e Instituto Brasileiro de Geografia e Estatística. Pesquisa Nacional por Amostra de Domicílios: acesso e utilização de serviços de saúde 2003. Rio de Janeiro; 2005. Available from: http://biblioteca.ibge.gov.br/visualizacao/instrumentos_de_coleta/doc0935.pdf

f Ministério da Saúde (BR), Secretaria de Atenção à Saúde, Departamento de Atenção Básica, Coordenação Geral de Saúde Bucal. SB Brasil 2010: Pesquisa Nacional de Saúde Bucal: projeto técnico. Brasília (DF); 2009. Available from: http://dab.saude.gov.br/CNSB/sbbrasil/arquivos/Projeto_SB2010_VersaoFinal.pdf

g Ministério da Saúde (BR), Secretaria de Atenção à Saúde; Secretaria de Vigilância em Saúde. SB Brasil 2010: Pesquisa Nacional de Saúde Bucal: resultados principais. Brasília (DF); 2012 [cited 2017 Jun 25]. Available from: http://bvsms.saude.gov.br/bvs/publicacoes/pesquisa_nacional_saude_bucal.pdf

h Ministério da Saúde (BR), Secretaria de Atenção à Saúde, Departamento de Atenção Básica. Envelhecimento e saúde da pessoa idosa. Brasília (DF); 2006 [cited 2017 Jun 25]. (Série A. Normas e Manuais Técnicos); (Cadernos de Atenção Básica, 19). Available from: http://189.28.128.100/dab/docs/publicacoes/cadernos_ab/abcad19.pdf
